# In-Ear Pulse Rate Measurement: A Valid Alternative to Heart Rate Derived from Electrocardiography?

**DOI:** 10.3390/s19173641

**Published:** 2019-08-21

**Authors:** Stefanie Passler, Niklas Müller, Veit Senner

**Affiliations:** Technical University of Munich, Department of Mechanical Engineering, Professorship of Sport Equipment and Materials, Boltzmannstraße 15, D-85747 Garching, Germany

**Keywords:** photoplethysmography, heart rate, consumer-wearable devices, in-ear, validation, optical pulse rate monitoring, pulse rate

## Abstract

Heart rate measurement has become one of the most widely used methods of monitoring the intensity of physical activity. The purpose of this study was to assess whether in-ear photoplethysmographic (PPG) pulse rate (PR) measurement devices represent a valid alternative to heart rate derived from electrocardiography (ECG), which is considered a gold standard. Twenty subjects (6 women, 14 men) completed one trial of graded cycling under laboratory conditions. In the trial, PR was recorded by two commercially available in-ear devices, the Dash Pro and the Cosinuss°One. They were compared to HR measured by a Bodyguard2 ECG. Validity of the in-ear PR measurement devices was tested by ANOVA, mean absolute percentage errors (MAPE), intra-class correlation coefficient (ICC), and Bland–Altman plots. Both devices achieved a MAPE ≤5%. Despite excellent to good levels of agreement, Bland–Altman plots showed that both in-ear devices tend to slightly underestimate the ECG’s HR values. It may be concluded that in-ear PPG PR measurement is a promising technique that shows accurate but imprecise results under controlled conditions. However, PPG PR measurement in the ear is sensitive to motion artefacts. Thus, accuracy and precision of the measured PR depend highly on measurement site, stress situation, and exercise.

## 1. Introduction

As a result of the development of mobile heart rate monitors, heart rate has become one of the most widely used methods for controlling the general state of health and, particularly, the intensity of physical activity [1]. In 1938, Hertzman [2] first introduced photoplethysmography (PPG) as an alternative to electrocardiographic (ECG) heart rate monitoring. Since then, PPG has gained increasing popularity [3] and, with increasing technological improvements, it is used as an alternative pulse rate measurement method in wearable devices. In his review article, Toshiyo Tamura [4] provides an overview of the parameters that influence PPG signals. Besides the wavelength of light, contact force, motion artefacts, ambient temperature, and light intensity, the anatomical measurement location also influences PPG signals. Currently, PPG signals can be measured at the wrist [5,6,7], upper and lower arm [8,9,10], finger [11], esophageal region [12], forehead [13,14], and the ear, respectively. Ear-worn devices are defined as devices worn in or on the ear. Specifically, pulse rate measurement at the earlobe [15], the external ear cartilage [16,17], the superior and inferior auricular region [18,19,20,21], and the external auditory canal [22,23,24] has already been discussed in several studies as an alternative to ECG heart rate monitoring. Besides wrist-worn devices, ear-worn devices are probably the most common application of PPG.

The measuring principle of a PPG sensor is based on optical variations in pulsatile blood-flow volume. A PPG sensor includes a light-emitting diode (LED) and a photodetector (PD). The arrangement of these two components determines the mode of PPG. There are two modes, transmission and reflectance. In transmission mode, the PD, located opposite to the LED, captures the light transmitted through the tissue. Especially in medicine, this mode is frequently used. In it, the finger is the most common measurement location. A clip enables the opposite positioning of LED and PD. However, blood flow in the extremities, e.g., in the fingers, can be severely impaired due to circulatory disorders, for example. This may lead to unreliable and invalid measurement of the pulse rate. In reflectance mode, the PD captures the light reflected from bony structures, tissue, and blood vessels. Moreover, LED and PD are located next to each other. Thus, the reflectance mode is hardly limited to certain anatomical measurement locations [25,26]. Obviously, this is a reason for the suitability of PPG’s reflectance mode in wearable devices. However, the intensity of the reflected or backscattered light is strongly dependent on the anatomical conditions of the measuring location [13]. On the forehead, where skin is very thin, but many blood vessels are present, a reliable signal can usually be recorded. On anatomical locations with a lower density of blood vessels and bony structures, the detected light intensity is usually lower. 

Common wavelengths of PPG are between 500 nm and 1100 nm. This corresponds to the range from green-yellow to infrared light. The absorption rate of the light is mainly influenced by the pigment melanin and the water content in the tissue. The correlation between melanin and the absorption rate can be described as inversely proportional; the longer the wavelength, the more light is absorbed by melanin [27]. Water absorbs light in the ultraviolet and upper infrared range, whereas red and near infrared (NI) light is slightly absorbed by water [28]. This shows that the spectrum from NI to infrared light should be used for measuring deeper tissue structures. Hence, the transmission mode of PPG often uses wavelengths of 500–600 nm. Because of the stronger plethysmography signal, the green spectrum of light is more suited for optical pulse rate measurement [9]. The use of green LEDs allows a more accurate detection of the pulse rate [9,29,30,31].

The main difference between heart rate and pulse rate is the duration the pulse wave takes to complete the distance from the heart to the measurement site. This time is named the pulse transit time (PTT). It is determined as the time lag between the peak of the R-wave on the ECG and the peak value of the corresponding pulse at the measurement site, measured by PPG.

Altogether, several studies show that PPG is a promising method with regard to its use in wearable devices, especially when applied on the ear. However, most of the above-mentioned studies evaluated non-commercial devices, applied on the ear. So far, to our knowledge, no comprehensive studies have been published on the validity of commercial in-ear pulse rate monitoring devices using PPG. Thus, detailed validation of in-ear pulse rate monitoring devices using PPG is still pending. 

The purpose of this study is to evaluate the validity of two consumer-wearable in-ear devices with respect to an ECG. Within the scope of this study, heart rate and PPG signals were first recorded simultaneously and then the rate variability of the two methods was compared.

## 2. Materials and Methods

### 2.1. Participants

Twenty healthy subjects (men = 14, women = 6) participated in the study and provided their written informed consent. Study protocol was proceeded under medical supervision of the Department of Prevention, Rehabilitation, and Sports Medicine of the Technical University of Munich. Additionally, the study was conducted in accordance with the Declaration of Helsinki. Participants who were eligible received detailed information on the purpose and methods of the study, as well as on data treatment and confidentiality according to the General Data Protection Regulation (2016/679) of the European Parliament and the Council of 27 April 2016 [32] and its Corrigendum of 23 May 2018 [33]. The characteristics of the sample population are shown in Table 1.

### 2.2. Instruments

Within the scope of this study, the validity of the Cosinuss°One and the Dash Pro in-ear pulse rate measurement devices was investigated. 

#### 2.2.1. The Dash Pro

**Figure 1:**
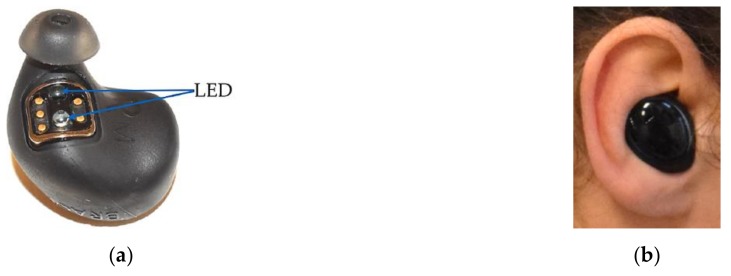
(**a**) The Dash Pro with silicone cap in size M; blue arrows show the LEDs for pulse rate detection. (**b**) Right Dash Pro, worn in the right ear.

The Dash Pro (Bragi, Munich, Germany) is a wireless headset, equipped with sensor technology that is able to provide real-time feedback of recorded movements and pulse rate. The device consists of left and right headphones that communicate wirelessly with each other. The Dash Pro measures pulse rate in the external auditory canal using infrared light by reflection measurement. The device itself is available in one size, but can be fitted to the user’s ear with interchangeable silicone caps in sizes XS to L. Figure 1a shows the Dash Pro with a silicone cap in size M. The blue arrows mark the diodes that allow heart rate detection by reflection measurement. Figure 1b shows the right device, worn in the right ear of a participant.

For the comparison of the two in-ear pulse rate measurement devices, they had to be worn simultaneously. Therefore, the right Dash Pro has a self-sufficient and independent single-use mode.

#### 2.2.2. Cosinuss°One

**Figure 2:**
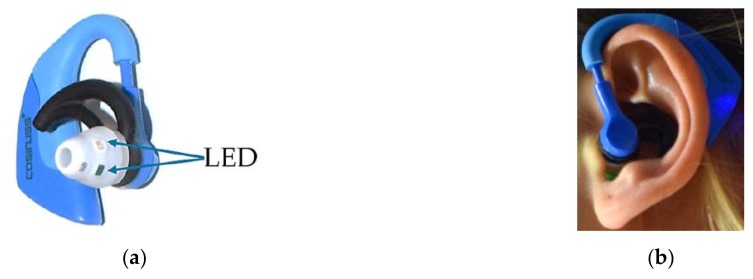
(**a**) Cosinuss°One; blue arrows show the LEDs for pulse rate detection. (**b**) Left Cosinuss°One, worn in the left ear.

The second in-ear pulse rate measurement device evaluated in this study was the Cosinuss°One (Cosinuss, Munich, Germany). For comparison with the Dash Pro, the Cosinuss°One was used in the participant’s left ear. Cosinuss°One measures the pulse rate in the external auditory canal by means of reflection measurement. Green light is used for this purpose. Measurement accuracy is ±1 bpm, as specified by the manufacturer. To optimize the fit and minimize movement artefacts, the device is available in sizes S to L. Individual size could be determined by means of the Cosinuss° app, using a value representing the received signal strength of the PO sensor. At a signal quality above 60%, size can be considered suitable. Figure 2a shows the Cosinuss°One. The two LEDs used for the reflection measurement in the external auditory canal are marked with blue arrows. Figure 2b shows the Cosinuss°One worn in the left ear of a participant.

#### 2.2.3. Criterion Measure ECG—Bodyguard 2

Bodyguard 2 (Firstbeat Technologies Oy, Jyväskylä, Finland) was used as criterion measure. This ECG is not a medical device, but a sports-oriented electrocardiogram suitable for long-term and exercise ECG. Heart rate is recorded with two electrodes and processed with an integrated algorithm to correct artefacts. Compared to a clinical standard ECG, the Bodyguard 2 indicates 99.98% agreement [34].

### 2.3. Experimental Protocol

In order to evaluate the two in-ear pulse rate measurement devices as comprehensively as possible, their validity was evaluated during rest and under stress on a bicycle ergometer (Excalibur sport, Lode, Groningen, The Netherlands). Protocol started with a 10-min rest measurement in lying position, followed by a standardized, self-designed exercise protocol in a controlled laboratory setting. Prior to measurement under stress, each participant was instructed to cycle at a stress of 50 W for a 3-min warm-up period. Measurement under stress then started. The stress pattern of the protocol depended on the subject and was calculated from his/her weight in order to achieve an appropriate, comparable increase in intensity and thus heart rate for each subject. The number of watts per kilogram of body weight per minute increased uniformly by 0.4 W and 0.3 W in male and female subjects, respectively, starting from 50 W. The aim of this test protocol was to record a stress phase of at least 10 min and to measure a heart rate range of approximately 100–170 bpm. In order to ensure that participants did not stop prematurely because of maximum exhaustion, the duration of the protocol was set to 20 min. Formulas (1) and (2) show the calculation of the individual target stress of male and female participants.

Target stress for males = 50 W + (weight × 0.4 × 20 min),(1)

Target stress for females = 50 W + (weight × 0.3 × 20 min).(2)

The participants were instructed to cycle at a self-chosen number of rounds per minute (rpm) up to the individual maximum stress and to stop by hand signal in case of exhaustion. Upon completion, participants cooled down by cycling at a stress of 50 W for 3 min. During the entire data recording, participants were instructed to speak as little as possible, since jaw movements can lead to movement artefacts [21]. 

Regarding the use of all devices, care was taken to follow user guidelines as suggested by the manufacturers. 

### 2.4. Data Analysis

ECG data were sampled at 1000 Hz and the heart rate was calculated from the time between R-R intervals, then exported as a text file at 1 s intervals. Data from optical pulse rate measurement of the in-ear devices was sampled at 100 Hz. The Cosinuss°One and the Dash Pro report the currently measured pulse rate to the respective mobile device app. For further analyses, data files of both in-ear devices were downloaded at 5 s intervals. Afterwards, data files of the in-ear and ECG devices were synched using the respective timestamps of each data acquisition. 

To ensure the synchronization of the in-ear and the ECG devices, their timestamps have to be reliable and identical. Therefore, all data files were recorded in the Unix timestamp format (UTC). This format counts time in milliseconds since 1 January 1970. In contrast, the Dash Pro counts time in milliseconds since 1 January 2015. This represents an overall time discrepancy of 45 years or a shift by up to 40 s within 24 h. This correction was carried out immediately before each examination.

Time-synched data from each device were concurrently and continuously acquired for each participant throughout the entire test protocol. In accordance to the validation study of Spierer et al. [31], a 5 s time interval was defined as sufficiently accurate for detecting significant variations in heart rate measurement. Hence, every fifth value of the heart rate was used for further analysis. 

**Figure 3:**
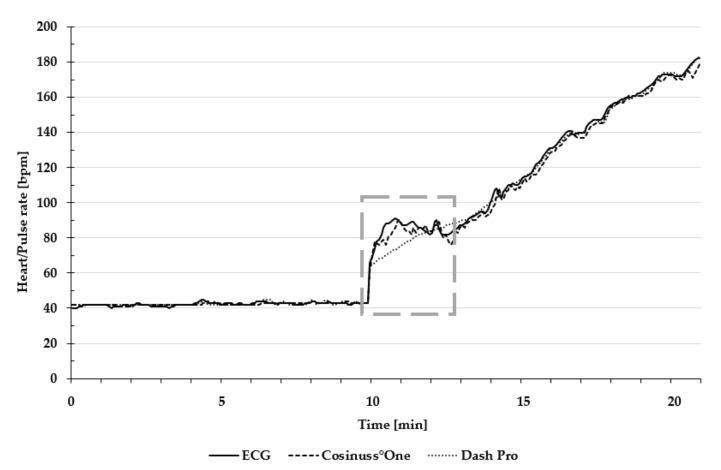
Exemplary presentation of pulse and heart rate during the entire test protocol. The dashed frame indicates the change from lying position to cycling. Heart rate of the ECG is depicted as a solid line; the pulse rate of Cosinuss°One is depicted as the dashed line; and the pulse rate of Dash Pro is depicted as the dotted line.

Figure 3 presents heart and pulse rate during the entire test protocol, including the change from lying position to cycling.

Motion artefacts, due to the change of body position and the re-adjustment of the sensors, resulted in strong signal noise, which can be seen in the dashed frame of Figure 3. Consequently, this data was not taken into account in the statistical evaluation.

Statistical analyses were conducted using IBM’s SPSS Statistics software version 24 (IBM, Armonk, NY, USA). Descriptive statistics were used to characterize the sample population. The validity of the in-ear pulse rate measurement devices was determined by means of several statistical tests. Looking more closely at the term of validity, a distinction should be made between accuracy and precision. In the present study, accuracy was tested by MAPE and ICC values, whereas precision was identified by the limits of agreement of the Bland–Altman analysis.

Mean absolute percentage errors (MAPE) compared to the criterion measure were calculated as indicators of measurement error. MAPE, representing the error as a percentage of the overall mean relative to the ECG, does not have a standardized threshold for determining the accuracy of measurements. In the present study, a MAPE of ≤5% [35] was used as the criterion value for accuracy. To further investigate the level of agreement, Bland–Altman plots [36] were prepared. These plots serve as a visual illustration of variance and over- or underestimated measurement ranges of the investigated in-ear devices. For this, limits of agreement were set to 95%. Maximum and minimum pulse rates measured by means of the in-ear devices were compared with the ECG results using one-way repeated-measures ANOVA. An alpha of 0.05 was used to determine statistical significance. In addition, the agreement of maximum rate between the ECG and the tested devices was defined by means of the Intra-Class Correlation Coefficient (ICC) according to Liu et al. [37]. Excellent, good, moderate, and low agreement thresholds were defined as ICC values of ≥0.90; 0.75–0.90; 0.60–0.75; and ≤0.60, suggested by Fokkema et al. [35].

## 3. Results

### 3.1. Preliminary Analysis

The Kolmogorov–Smirnov test as well as visual data plotting of the criterion measure (Bodyguard 2), Cosinuss°One, and the Dash Pro revealed that the overall rates among all participants were significantly different from a normal distribution (resting conditions: D(2376) = 0.066, *p <* 0.001 for ECG and D(2376) = 0.080, *p <* 0.001 for Cosinuss°One and D(2376) = 0.081, *p <* 0.001; stress conditions: D(2547) = 0.079, *p <* 0.001 for ECG and D(2547) = 0.075, *p <* 0.001 for Cosinuss°One and D(2547) = 0.073, *p <* 0.001 for Dash Pro). To account for the differences between criterion measure and alternative method, Bland and Altman [38] suggest to investigate the variances for normal distribution, too. The preliminary applied Kolmogorov–Smirnov test on differences between criterion measure and alternative method did significantly deviate from a normal distribution. However, visual inspection indicated mostly normally distributed data (resting conditions: D(2376) = 0.172, *p <* 0.001 for Cosinuss°One and D(2376) = 0.192, *p <* 0.001 for Dash Pro; stress conditions: D(2547) = 0.280, *p <* 0.001 for Cosinuss°One and D(2547) = 0.216, *p <* 0.001 for Dash Pro). Hence, the procedure suggested by Bland and Altman [38,39] was implemented. 

### 3.2. Resting Heart/Pulse Rate and Heart/Pulse Rate ≤90 bpm

The differences between the resting rates and the rates ≤90 bpm of the investigated in-ear devices and the ECG are provided in Table 2. 

On average, the participants achieved a resting heart rate of 54.9 ± 10.1 bpm in the ECG examinations. The average in-ear measured resting pulse rate of the Cosinuss°One and the Dash Pro is 53.6 ± 8.3 bpm and 55.0 ± 9.7 bpm, respectively.

**Figure 4:**
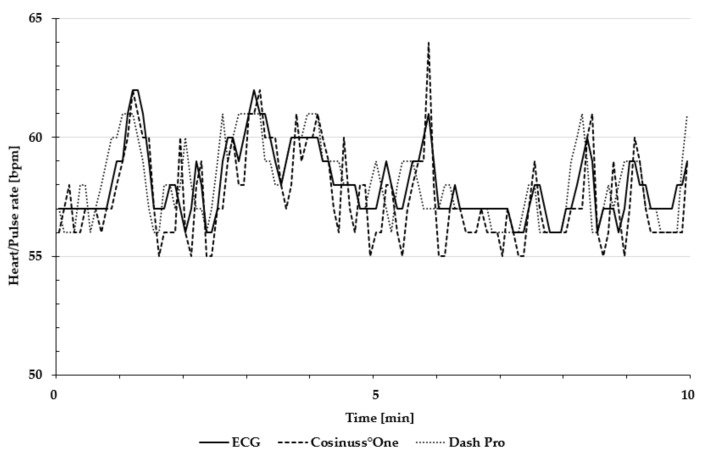
Exemplary presentation of pulse and heart rate during the 10 min rest measurement in a lying position. The heart rate of ECG is depicted as the solid line; the pulse rate of Cosinuss°One is depicted as the dashed line; and the pulse rate of Dash Pro is depicted as the dotted line.

Figure 4 is an exemplary presentation of the in-ear devices’ pulse rate and the ECG’s heart rate, recorded during the 10 min rest measurement in a lying position.

For the main analysis, one-way repeated-measures ANOVA was conducted. Mauchly’s tests indicated that the assumption of sphericity had not been violated, X²(2) = 4.27, *p >* 0.05, therefore non-corrected tests are reported. The results revealed that the minimum rates were not significantly affected by the measurement device, F(2, 38) = 3.17, *p >* 0.05. 

Both in-ear devices are quite similar in terms of mean absolute error and MAPE, whereas the Dash Pro indicates higher values than the Cosinuss°One. In addition, the Cosinuss°One and the Dash Pro show excellent agreement in comparison to ECG, *R* = 0.94 for Cosinuss°One and *R* = 0.98 for Dash Pro.

**Figure 5:**
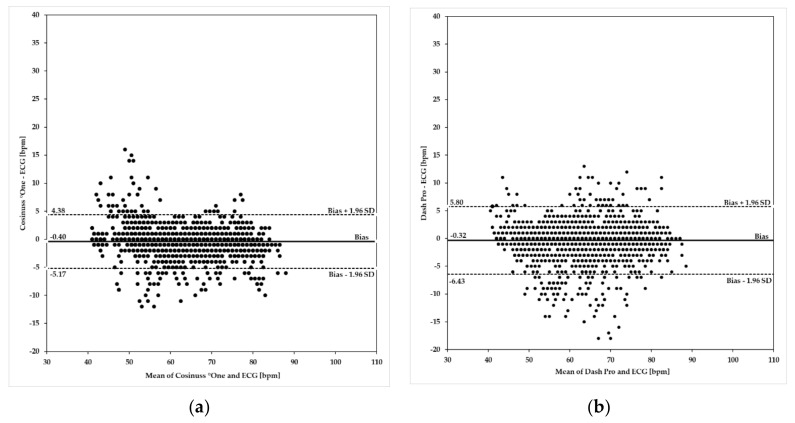
Bland–Altman plots using heart/pulse rates ≤90 bpm. Investigated in-ear devices: (**a**) Cosinuss°One; (**b**) Dash Pro. Plots indicate differences of the rate values on the y-axis relative to the mean of the two methods (ECG and in-ear measurement) on the *x*-axis. Limits of agreement (LoA) were calculated as mean ± 1.96 × SD. Biases are depicted as a solid line; LoA are depicted as dashed lines.

Figure 5 shows Bland–Altman plots of the Cosinuss°One and the Dash Pro in-ear devices in comparison to the ECG with Bodyguard 2. 

Upper and lower limits of agreement (ULoA, LLoA) as well as the mean differences of the in-ear devices’ PR ≤90 bpm compared to the ECG’s HR are labeled in Figure 5a,b. Both in-ear devices show considerable deviations in scattering when compared to the ECG and tend to underestimate the heart rate by 0.40 bpm for Cosinuss°One and by 0.32 bpm for the Dash Pro. Bland–Altman analysis of the Cosinuss°One (Figure 5a) and the Dash Pro (Figure 5b) show that variability occurred across the spectrum of rates ≤90 bpm. The Cosinuss°One indicates more variability between 40 bpm and 60 bpm, while the Dash Pro shows higher variability across the midrange rates. In addition, the differences in variance are visualized. The Cosinuss°One (ULoA-LLoA: 9.55 bpm) shows lower scattering among its measurements when compared to the Dash Pro (ULoA-LLoA: 12.23 bpm). The Cosinuss°One had 95% of differences within +4.38 bpm and −5.17 bpm of the ECG, while the Dash Pro had 95% of differences within +5.80 bpm and −6.43 bpm.

### 3.3. Heart/Pulse Rate ≥100 bpm

The mean absolute error and the MAPE of the investigated in-ear devices’ pulse rate ≥100 bpm are provided in Table 3.

**Figure 6:**
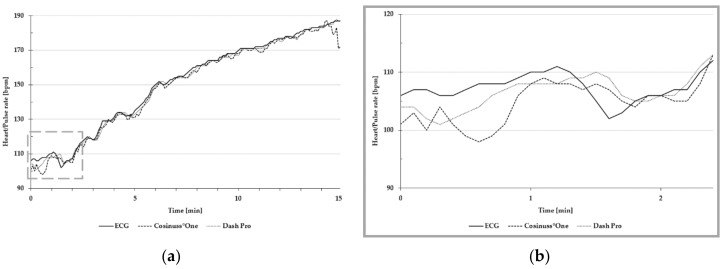
Exemplary presentation of pulse and heart rate (**a**) during the entire stress protocol; (**b**) around 100 bpm. The heart rate of ECG is depicted as the solid line; the pulse rate of Cosinuss°One is depicted as the dashed line; and the pulse rate of Dash Pro is depicted as the dotted line.

Both in-ear devices are quite similar in terms of mean absolute error and MAPE. Figure 6a is an exemplary presentation of the in-ear devices’ pulse rate and the ECG’s heart rate, recorded during the stress protocol to the individual exhaustion. Figure 6b illustrates high differences between ECG and the in-ear devices around 100 bpm.

**Figure 7:**
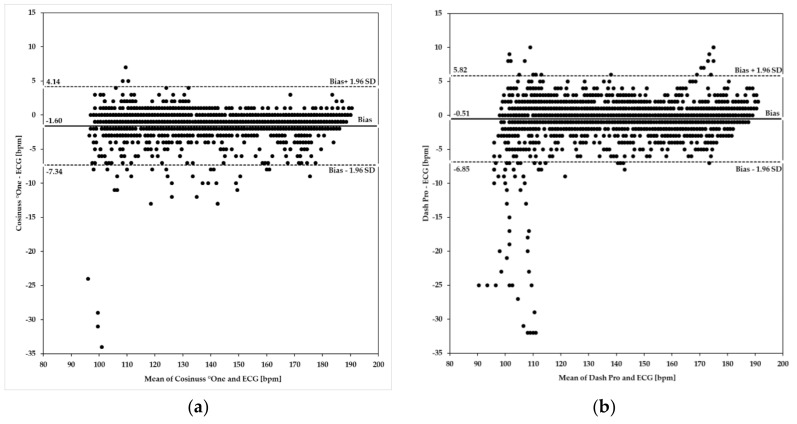
Bland–Altman plots using heart/pulse rate ≥100 bpm. Investigated in-ear devices: (**a**) Cosinuss°One; (**b**) Dash Pro. Plots indicate differences of the rate values on the y-axis relative to the mean of the two methods (ECG and in-ear measurement) on the x-axis. Limits of agreement (LoA) were calculated as mean ± 1.96 × SD. Biases are depicted as a solid line; LoA are depicted as dashed lines.

Figure 7 shows Bland–Altman plots of the Cosinuss°One and the Dash Pro in-ear devices in comparison to the ECG with Bodyguard 2.

Upper and lower limits of agreement (ULoA, LLoA) as well as the mean differences of the in-ear devices’ PR ≥100 bpm compared to the ECG’s HR are labeled in Figure 7a,b. Both in-ear devices show considerable deviations in scattering when compared to the ECG and tend to underestimate heart rate by 1.60 bpm for Cosinuss°One and by 0.51 bpm for the Dash Pro. Bland–Altman analysis of the Cosinuss°One (Figure 7a) and the Dash Pro (Figure 7b) shows that variability occurred across the spectrum of rates ≥100 bpm. The Cosinuss°One indicates more variability between 100 bpm and 150 bpm, while the Dash Pro shows higher variability between 100 bpm and 115 bpm. In addition, the differences in variance are visualized. The Cosinuss°One (ULoA-LLoA: 11.48 bpm) shows lower scattering among its measurements when compared to the Dash Pro (ULoA-LLoA: 12.67 bpm). The Cosinuss°One had 95% of differences within +4.14 bpm and −7.34 bpm of the ECG, while the Dash Pro had 95% of differences within +5.82 bpm and −6.85 bpm.

### 3.4. Maximum Heart/Pulse Rate 

The differences between the maximum heart/pulse rate of the investigated in-ear devices and the ECG are provided in Table 4. 

On average, participants achieved a HR_max_ of 183.0 ± 5.1 bpm in the ECG examinations. The average in-ear measured PR_max_ of the Cosinuss°One and the Dash Pro is 181.6 ± 6.4 bpm and 183.7 ± 4.8 bpm, respectively. 

For the main analysis, one-way repeated-measures ANOVA was conducted. Mauchly’s test indicated that the assumption of sphericity had not been violated, X²(2) = 3.66, *p >* 0.05, therefore non-corrected tests are reported. In addition, the results revealed that the maximum rates were significantly affected by the measurement device, F(2, 38) = 3.80, *p ≤* 0.05. In addition, the Cosinuss°One and the Dash Pro show good agreement in comparison to ECG, *R* = 0.84 for Cosinuss°One and *R* = 0.83 for Dash Pro.

### 3.5. Motion Artefacts

**Figure 8:**
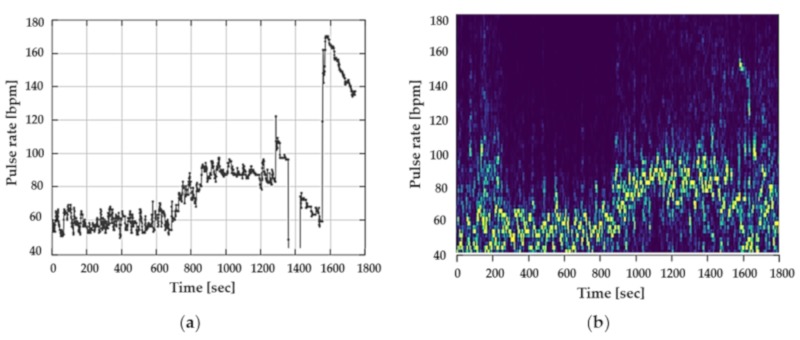
Influence of jaw movements and talking on the PPG signal of both in-ear devices: (**a**) Cosinuss°One; (**b**) spectrogram of PPG signal of the Dash Pro.

To illustrate the influence of jaw movements on the PPG signal, one participant was asked to chew chewing gum as well as to talk throughout the data recording. The effects on the PPG signal and the pulse rate can be clearly seen in Figure 8a,b. Figure 8a illustrates the pulse rate of the Cosinuss°One, Figure 8b shows the spectrogram of the PPG signal of the Dash Pro.

Both figures depict the significant influence of motion on the PPG signal and the effect on the pulse rate. In both devices, signal interference was too intense to determine a precise pattern of the pulse rate.

## 4. Discussion

The present study examined the accuracy and precision of two ear-worn pulse rate measurement devices using PPG technology. 

Systematic differences should be assessed using the MAPE. According to Nelson et al. [40], wearable devices should not exceed a MAPE threshold of ≤10% in order to be considered accurate. Fokkema et al. [35] suggest a threshold of ≤5%. In the present study, both devices indicated smaller MAPE scores at rates ≥100 bpm than at rates ≤90 bpm, but not exceeding the threshold of 5%. Thus, both in-ear devices can be classified as accurate almost completely within heart rates of 60–190 bpm, even according to Fokkema et al. [35]. 

To investigate the level of agreement between the in-ear devices and the criterion measure ECG, Bland–Altman plots were prepared according to Bland and Altman [36]. In resting condition as well as within the range of pulse rates ≥100 bpm, the Cosinuss°One revealed a narrower 95% limit of agreement than the Dash Pro. Both devices tended to slightly underestimate heart rate values within 60–190 bpm and showed high deviations from the ECG around 100 bpm. Although the limits of agreement might seem pretty narrow, these results have to be considered carefully. Heart and pulse rate data are a very sensitive source, e.g., for app-based training programs, and therefore imprecise and inaccurate raw data can lead to fatal results.

To determine the level of agreement, the ICC values of resting heart and pulse rates as well as maximum heart and pulse rates were examined. Under resting conditions, the Dash Pro and the Cosinuss°One show excellent agreement in comparison to ECG, whereas at maximum heart rates, a decrease to good agreement of both devices could be observed. Despite not exceeding the MAPE threshold of ≤5% and good to excellent levels of agreement, the results revealed that the maximum rates were significantly affected by the measurement device. 

From a scientific point of view, there is only one commercial in-ear device, Bose Sound Sport (BSS; Bose Corporation, Framingham, MA, USA), which has been validated with respect to PPG pulse rate measurement to date. This underlines the lack of scientific validation studies. Pulse rate is a physiological parameter used for training control in sports or for monitoring the general state of health. Consumers must be protected, especially if activity trackers are to be used increasingly in the health and fitness sector. However, this access can only be defended or considered responsible if the current lack of transparency of the activity trackers industry is remedied through high-quality research, which can also help define general standards for these devices. The present study contributes to fill this gap. It shows the potential and the weaknesses of two commercially available in-ear devices in terms of measuring pulse rate.

Boudreaux et al. [41] conducted a study very similar to the present research. It is the first and, so far, the only study that investigated the accuracy and precision for pulse rate sensors in commercial headphones. The difference of this to the present study is the measurement site of PPG pulse rate detection. The measurement site of the Bose Sound Sport is the auricle. Both in-ear PPG devices validated in the present study use the external auditory canal for pulse rate measurement. Considering the measurement site and the consumer market, it should be noted that the present study is the first one to validate commercially available pulse rate monitors using PPG in the external auditory canal.

In the study of Boudreaux et al. [41], eight wearable devices, including the ear-worn Bose Sound Sport PPG device, were compared to a six-channel ECG regarding heart rate and caloric expenditure measurements. In addition to other stress situations, the BSS was also validated during cycling. For this purpose, a graded stress protocol was used, starting at rest and ending at a maximum stress of 200 W. The BSS slightly overestimates heart rate during low cycling intensities and underestimates heart rate during higher intensities. The latter agrees with the results of the present study. On average, the MAPE of the BSS was 7.4%. Under resting conditions, the MAPE of the BSS was 3.2%, very close to the Dash Pro and the Cosinuss°One, with MAPE values of 3.2% and 2.5%, respectively. 

Considering the level of agreement, Boudreaux et al. [41] observed decreasing ICC values with higher exercise intensity and higher heart rates, respectively. This is in line with the results of the present study. The Cosinuss°One and the Dash Pro indicate a decrease from excellent to good agreement.

On the basis of MAPE values ≤5% and excellent to good levels of agreement, it can be stated that both the Dash Pro and the Cosinuss°One deliver accurate pulse rate values. In addition to accuracy, precision must also be considered as key factor when testing the validity of wearable devices. Due to high variance measured over the entire spectrum of heart/pulse rates, both in-ear devices of the present study have to be considered too imprecise as to be used as an alternative to the ECG.

Other than the mentioned study of Boudreaux et al. [41] and the present research, where commercially available ear-worn PPG devices were tested, the studies of Tigges et al. [23], Budidha and Kyriacou [16], and Leboeuf et al. [42] investigated self-developed pulse rate measurement systems based on PPG-technology. These devices are not commercially available. Tigges et al. [23] built such a device for scientific purposes and tested the device in the spectrum of 50–125 bpm under resting conditions. The Bland–Altman analysis showed a bias of −0.03 bpm, with 95% of the data lying within the boundaries of +2.88 bpm and −2.94 bpm. The study of Leboeuf et al. [42] showed a bias of −0.2 bpm, with the sensor slightly underestimating heart rate data. Similar to these results, where the data showed excellent agreement up to 200 bpm within varying activities, Budidha and Kyriacou [16] also concluded that the ear canal might be a suitable site for pulse rate measurement conducted by PPG sensors. 

In comparison to the in-ear pulse rate PPG devices, considerably more wrist-worn pulse rate devices were validated [7,43,44,45,46,47,48,49,50,51,52,53]. Both fitness trackers and sports watches were tested under different exercise conditions. In order to ensure comparability to the presented results of the in-ear devices, only the literature in which validation was conducted on both a bicycle ergometer and an ECG was used as criterion measure will be discussed further. Within the framework of these studies [43,44,45,46], the validity of the wrist-worn devices, e.g., Apple Watch, Fitbit Charge HR, Basis Peak, Samsung Gear S, Polar M600, was mostly examined at rest, low, and high intensities. All devices tended to underestimate the heart rate regardless of intensity under both resting and cycling conditions. For instance, Wallen et al. [43] and Horton et al. [46] indicated that increasing physical effort leads to decreasing accuracy of the heart rate measurement. The results of Wallen et al. [43] revealed that heart rate underestimation ranged from −0.52 bpm (Basis Peak) to −12.67 bpm (Fitbit Charge HR) at low intensities, and −7.42 bpm (Basis Peak) to −14.20 bpm (Fitbit Charge HR) at high intensities, depending on the wrist-worn device used. Horton et al. [46] compared the sports watch Polar M600 pulse rate with the ECG heart rate during rest and cycling. They demonstrated a heart rate underestimation of −0.1 bpm during rest and −1.9 bpm during cycling. These findings are in agreement with the present results that show heart rate underestimation ranged from −0.32 bpm (Dash Pro) to −0.40 bpm (Cosinuss°One) under resting conditions and from −0.51 bpm (Dash Pro) to −1.60 bpm (Cosinuss°One) under cycling conditions.

The results presented in this study suggest the weakness of the PPG pulse rate monitoring devices, both in-ear and the wrist-worn.

PPG is a low-cost medical technique applied to the skin that uses the transmission and reflection of light into the skin to measure changes in blood volume within a specific tissue [9]. Previous research [1,4,54] suggests that PPG devices may have limitations in measuring PR that arise from the continuous increase and decrease of compression of a wearable device’s PR sensor on the skin. In addition to conditions of low blood circulation and movements of the sensor on the skin, ambient light, which falsifies the values of the receiving diode, can also be seen as a source of error in PPG heart rate measurement [55]. In-ear PPG pulse rate monitors were developed to solve these main problems. As Vogel et al. [21] were able to show, a PPG sensor in the auricle near the ear canal, which irradiates light into the skin by reflection measurement, is resistant to most of the above-mentioned interfering factors. Circulatory disturbances and ambient light were minimized by the placement inside the ear. The inner ear is supplied by the same artery as the brain, which is why the blood flow and thus the strength of the PPG signal received in the inner ear remains constant, in contrast to PPG sensors on the extremities [16]. 

Due to pulse transit time, differences between heart rate and pulse rate may occur in general. Time lag between the peak of R-wave on the ECG and the peak value of the corresponding pulse in the ear, measured by PPG, may be a reason for underestimating heart rate. 

A main remaining weakness of PPG is an unreliable signal due to motion artefacts. For in-ear PPG pulse rate measurement, jaw movements are the most dominant artefact [21]. In the present study, the external auditory canal as an application site for commercially available PPG pulse rate measurements was therefore tested as an alternative to electrocardiographic heart rate measurement. 

While it was shown that the external auditory canal is a limited alternative measurement site for pulse rate measurement, the specific conditions in this study have to be taken into consideration. The study was conducted under controlled testing conditions, as the subjects were instructed to reduce jaw movement, e.g., by talking as little as possible to deliberately minimize motion artefacts. In addition, motion artefacts, such as head movement and vibrations due to uneven terrain, were not taken into account in this study. Despite the instruction to cycle at a self-chosen number of rounds per minute (rpm), most of the participants cycled at 80–100 rpm. This frequency may have increased the deviation of both of the in-ear devices from the ECG at a heart rate around 100 bpm. 

In his review article, Toshiyo Tamura [4] suggests various methods to eliminate motion artefacts. 

A conclusion regarding the higher validity of PPG due to different wavelengths when comparing both devices in the present study could not be made. Even though the Cosinuss°One measures with green light while the Dash Pro uses infrared light, significant differences in the measurement accuracy and precision could not be shown, opposing the suggestion that the use of green LEDs allows a more accurate and precise detection of the pulse rate [9,29,30,31].

The subject group represents just a certain and small population. Due to the young age and the healthy condition of the subjects, conclusions that consider older or diseased people cannot be drawn. In addition, a gender-specific differentiation of the results cannot be made due to the unbalanced distribution of fourteen men and six women. 

## 5. Conclusions

It can be concluded that PPG measurement in the ear is a promising technique in resting positions. For future improvement, however, motion artefacts should be significantly reduced in order to ensure accuracy and precision in in-ear PPG pulse rate measurement during activity. Therefore, extensive studies under real life conditions should be carried out for a better understanding of the interaction between measurement accuracy as well as precision and the effects of artefacts from motion. This requires a considerable contribution from the activity trackers industry. The existing lack of transparency, e.g., regarding the algorithms used, complicates the analysis of the problems and thus the development of solutions.

The results of this study give an insight into the validity of the PPG technology used in the ear. Particularly in consumer wearable devices, a device worn in the ear can have many advantages over chest- or wrist-worn devices, as sports-specific parameters could be broadcasted to the athlete without interfering with the current activity. The combination of hearing aids and their ability to measure pulse rate simultaneously, could be a future application of in-ear PPG technology to promote the safety of elders. For use in health care, general standards and guidelines, with respect to measurement accuracy and precision, have to be defined and introduced to the activity trackers industry.

## Figures and Tables

**Table 1:** Participant characteristics. Values are means ± standard deviation.

	Male (*N* = 14)	Female (*N* = 6)	All Participants (*N* = 20)
Age (years)	22.1 ± 1.8	22.5 ± 2.1	22.3 ± 2.0
Body mass (kg)	74.6 ± 8.4	57.8 ± 4.2	69.6 ± 11.0

**Table 2:** Comparison among different devices for resting heart/pulse rate and heart/pulse rate ≤90 bpm. Values are mean ± standard deviation (SD), intra-class correlation coefficient (ICC), mean absolute error (MAE) ± standard deviation (SD), and mean absolute percentage error (MAPE).

	Resting Heart/Pulse Rate		Heart/Pulse Rate ≤90 bpm	
	Mean ± SD (bpm)	ICC	MAE ± SD (bpm)	MAPE (%)
Cosinuss°One	53.6 ± 8.3	0.94	1.5 ± 1.8	2.5
Dash Pro	55.0 ± 9.7	0.98	2.0 ± 2.5	3.2
ECG	54.9 ± 10.1			

**Table 3:** Comparison among in-ear devices for pulse rates ≥100 bpm. Values are mean absolute error (MAE) ± standard deviation (SD), mean absolute percentage error (MAPE).

	Pulse Rate ≥ 100 bpm	
	MAE ± SD (bpm)	MAPE (%)
Cosinuss°One	1.8 ± 2.8	1.3
Dash Pro	1.8 ± 2.8	1.4

**Table 4:** Comparison among different devices for maximum heart/pulse rate. Values are mean ± standard deviation (SD), intra-class correlation coefficient (ICC).

	Maximum Heart/Pulse Rate	
	Mean ± SD (bpm)	ICC
Cosinuss°One	181.6 ± 6.4	0.84
Dash Pro	183.7 ± 4.8	0.83
ECG	183.0 ± 5.1	
